# Quality of Life and Conformity to Gender Norms in Women Receiving Assisted Reproductive Technologies as a Potential Indicator of Mental Health

**DOI:** 10.3390/ijerph191610031

**Published:** 2022-08-14

**Authors:** Lidia Bueno-Sánchez, Tamara Alhambra-Borrás, Alfonso Gallego-Valadés, Jorge Garcés-Ferrer

**Affiliations:** Polibienestar Research Institute, University of Valencia, 46022 Valencia, Spain

**Keywords:** perinatal mental health, infertility, assisted reproductive technology, gender norms, CFNI, quality of life, FertiQol

## Abstract

The prevalence of depression, stress, or anxiety in people receiving assisted reproductive technologies (ART) has been demonstrated. However, knowledge about the influence of gender norms on quality of life (QofL) during infertility treatment is limited. The main objective of this study was to confirm that patients undergoing ART present a vulnerable mental state, which may be an indicator of risk. For this purpose, a quasi-experimental cross-sectional study was carried out in the Assisted Reproduction Unit of the Hospital Politécnico Universitario de la Fe (Spain) in which a total of 438 women participated: 256 in pre-treatment and 182 in treatment. Two questionnaires were administered, FertiQol and CFNI-23, assessing self-perceived QofL and conformity to gender norms, respectively. The results showed significant differences between the pre-treatment and treatment groups on the FertiQol and its subscales. Significant associations were also found between the CFNI-23 factors and the FertiQol subscales. The results suggest that gender norms and ART interfere with women’s mental health and QofL and should be considered as possible risk indicators by professionals preventively before the prenatal or perinatal stages. Future research should design prospective studies aimed at estimating the impact of clinical and sociodemographic variables on women and other groups receiving ART.

## 1. Introduction

The European IVF Monitoring Consortium (EIM) for the European Society of Human Reproduction and Embryology (ESHRE) estimates that approximately 8 million babies have been born in Europe using assisted reproductive technologies (ART) [1]. Subfertility procedures are increasing across Europe, and policies related to their use are being developed in all countries; Spain, Belgium, Denmark, and the Czech Republic lead in the number of ART [2]. The increased use of human reproductive treatments and their effects on the mental health of patients are a public health issue to be considered. This is of particular concern for women, who are the main patients of treatments that are often long-term, intrusive, painful, and cyclical processes. The delay between artificial insemination or in vitro fertilization and the confirmation of pregnancy is one of the most distressing time periods [3,4,5]. In addition, in most cases, there is prior “baggage” full of stress and emotional burnout due to not being able to achieve pregnancy naturally and the impact of infertility itself [6,7,8,9]. Therefore, the woman’s quality of life (QofL) during and after treatment may be affected. This suggests a continuing need for sex–gender studies that allow a rigorous approach to the facts about the condition of women ART patients. Indeed, as has been evidenced, QofL during ART treatments will be influenced by preconceptions or expectations about the construction of motherhood and assumed gender norms [6,7,8,9].

Studies conducted over the last years have shown that social moderators (modulators) such as demographics, culture, or gender directly influence the social construct of childbearing and, thus, its channelling through laws, economic and social policies, health practices, etc. Gender norms are increasingly recognized as affecting the health and well-being of individuals [5,6,7,8,9]. WHO notes that “gender-related determinants of health are the social norms, expectations and roles that increase rates of exposure and vulnerability to, and protection from, health risks” [10]. The study of masculine and feminine gender norms and how they guide people’s behaviour is of particular importance, as adherence to these norms has been linked to a wide range of psychological, social, and health problems worldwide [5,6,7,8,9]. Studies support the idea that women who identify as such, and who remain in contexts where parenting is culturally and traditionally linked to women, score worse on QofL in infertility situations. In this context, women may be more preoccupied with the inability to conceive than men, making it a central fact of their lives and more present in their thoughts [11,12,13,14]. In contrast, other studies on the infertile population confirm that women who conform more to gender norms close to motherhood report better health [15].These studies also confirm that men who are more compliant with male gender norms report worse physical, psychological, and couple health [16].

Social constructs and cultural influences may explain the variability in how individuals and couples experience infertility and ART [16,17]. Knowledge of QofL and adherence to gender norms and/or expectations about childbearing during ART may be a basis from which to support families in their mental health before mental illness develops postnatally [18,19,20,21]. For this reason, the aim of this study was to explore the consistency of conformity to gender norms of women participating in ART processes and to analyse whether the degree of conformity modulates women’s quality of life before and during ART.

## 2. Materials and Methods

A cross-sectional comparative study was conducted with the aim of analysing the psychosocial variables assigned to gender roles and infertility-related quality of life of heterosexual women during ART pretreatment and treatment, attended with their partner. Data collection was conducted between January 2018 and August 2020 at the Assisted Reproduction Unit of the Hospital Universitario y Politécnico (HUP) La Fe in Valencia (Spain). All women who participated in this study provided written informed consent. The design and administration of the informed consent was approved by the ethics committee of the HUP La Fe. The participants included in the study completed the self-administered questionnaires in a physical space set up in the Assisted Reproduction Unit, administered by a member of the research team and delivered on the same day.

### 2.1. Data Management Plan and Confidentiality

Data Management Plan (DMP) was developed to securely managed the data between the members of the research team from the University of Valencia and those from the Hospital Politécnico Universitario de La Fe, where the sample was recruited. This DMP corresponds to in accordance with the terms required by the European Regulation (EU) 2016/679 regarding the processing of personal data [22]. The following aspects included:

- Access was restricted under the supervision of the coordinator of the Assisted Reproduction Unit to the clinical history data.
- (Pseudo) Anonymization of identification and clinical history data.
- An adequate room was provided to administer the FertiQol and CFNI-23 questionnaires in person to the patients, ensuring their privacy.
- The questionnaires in physical format and the informed consent forms were kept in the facilities of the University of Valencia for this purpose, with restricted access under administrative and registry authorization.
- The electronic data were stored in a trusted repository provided by the University of Valencia (https://disco.uv.es/ (accessed on 1 May 2022)).
- Finally, following FAIR principles, the data were made available to the scientific community under open license in the Mendeley Data repository (see Data Availability Statement section).

### 2.2. Sample Selection

The sample size was calculated for a finite population with a confidence level of 95% and a margin of error of 5% for 1300 women in pretreatment and for 400 women in treatment. It was selected by convenience strictly to exclude other intervening variables (previous treatment, single-parent families, etc.). This study involved cisgender persons born with a female reproductive tract and who self-identify as woman. The sample consisted of a total of 438 women divided into two groups: women attending ART for the first time (*n* = 256) and women already receiving treatment (*n* = 182). The pretreatment group corresponds to women who were first-time visitors to the ART Unit referred by the Primary Health Care Centers. These were women who had never undergone infertility treatment. The women in the in-treatment group were those who were already receiving infertility treatment at that hospital.

The dataset has missing values related to both clinical and socio-demographic variables as well as questionnaire scores. It has been taken into account that clinical and socio-demographic variables are not substitutable, and that measures of substitution of missing values in questionnaires have an impact on data dispersion. Therefore, only subsamples of women with available data for the involved variables were included in each analysis. Degrees of freedom (df) are specified in parentheses for each of the summary statistics of the reported differences.

### 2.3. Inclusion Criteria

The inclusion criteria for the study were: (i) women over 18 years of age; (ii) women suffering from primary infertility; (iii) women up to 40 years of age at the start of treatment; (iv) heterosexual women accompanied by their partners; (v) women attending ART for the first time; (vi) women who are in some phase of treatment; (vii) women who know the Spanish language in writing.

### 2.4. Study Variables

Two variables were studied: quality of life and gender norm conformity. To measure quality of life, participants were administered the Quality of Life in Fertility Questionnaire (FertiQol) [23]. The questionnaire was developed jointly by the European Society of Human Reproduction and Embryology (ESHRE) and the American Society of Reproductive Medicine (ASRM) to assess the quality of life of people experiencing infertility. The FertiQol questionnaire has been validated and translated into 46 languages, available on the Cardiff University website [24]. The Spanish version of the questionnaire was requested for this study. FertiQol assesses the impact of infertility problems in several areas: personal quality of life, interpersonal quality of life, treatment-related quality of life, and overall satisfaction with physical health and quality of life. The instrument consists of 24 items, divided into two modules: a general module and a treatment module. The general module assesses quality of life in 4 subscales: emotional (feelings and individual experiences associated with fertility problems such as depression or envy), mind–body (physical and cognitive symptoms such as lack of concentration or tiredness), relational (aspects related to the relationship with a partner), and social (measures the impact on social interactions, support, etc.). The treatment module assesses the perception of treatment in two subscales: treatment environment and treatment tolerability. All items are scored on a Likert scale from 0 to 4, with a higher value corresponding to a higher quality of life. The range of final scores is 0–100. A higher score translates into a higher quality of life [23,25].

To measure gender norm conformity, participants were administered the adapted Spanish version of the Female Gender Norms Conformity Inventory (CFNI-23) [26,27]. It is an instrument that allows for measuring different aspects of femininity, as it integrates a response method based on behaviours, affects, and cognitions that allows us to assess from a broad perspective the ways in which women adhere to a norm. The CFNI-23 consists of a 4-point Likert-type scale ranging from 0 (strongly disagree) to 3 (strongly agree). The total score of the questionnaire can range from 0 to 69. The higher the score, the higher the agreement. The questionnaire assesses conformity with seven female gender norms: care for children, investment in appearance, domestic aspects, sexual fidelity, relational aspects, thinness, and modesty. Values for both scales ranged from 0 to 0.61 (CFNI-23) and 0.89 (FertiQol). Descriptive analyses were also performed for sociodemographic variables (Table 1).

### 2.5. Statistical Analysis Performed

The statistical analysis reported in the Section 3 consists mainly of: (1) measuring the correlations between the subscales of both instruments and between the total score and the variable number of infertility treatment cycles; and (2) contrasting the differences in the score of both instruments between different groups delimited by clinical variables and treatment status (pretreatment (PRE) vs. treatment).

The Lilliefors-corrected Kolmogorov–Smirnov test indicated that the FertiQol (*p* = 0.293) and CFNI-23 (*p* = 0.315) scores followed a near-normal distribution in the PRE group, but not in the Treatment group (*p* = 0.014 and *p* = 0.018, respectively). For this reason, Spearman’s correlation statistic was used to measure the association between subscales in this group, and Pearson’s r was used in the PRE group.

The *t*-test for unpaired samples was used to assess the contrast between two means. Welch’s correction was used in cases where homogeneity of variances could not be assumed, based on the results of a variance ratio pretest. For testing means among more than two groups, one-way ANOVA was implemented.

## 3. Results

### 3.1. Sample Profile

The mean age of the women was 34.85 years old. Most of the participants were of Spanish nationality (88.3%), followed by other non-European and European nationalities, representing 6.0% and 5.7%, respectively. Almost half of the women surveyed were married at the time of questionnaire administration (45.5%). The predominant educational level was university studies, representing 47.5% of the total sample. The majority of respondents were employees (79.2%).

### 3.2. Results of the Female Gender Norms Conformity Inventory (CFNI-23) and Quality of Life in Fertility Questionnaire (FertiQol)

Analyses showed no significant differences between the groups in terms of the total CFNI-23 values; *t*(314) = 0.05, *p* = 0.96. However, there were significant differences in the FertiQol total score between both groups; *t*(225) = 2.266, *p* = 0.024. The mean of the PRE group was higher than that of the Treatment group. Table 2 shows the differences between the groups on the FertiQol and CFNI-23 questionnaires.

There were significant differences in the mean scores of the FetiQol subscales between the groups. The PRE group scored higher than the Treatment group on all FetiQol subscales except for the Relational subscale. In CFNI-23, women in PRE had a higher mean score in the Childcare factor (M = 10.12, SD = 1.90) compared to the Treatment group (M= 9.53, SD = 2.41). No statistically significant differences were found in the other factors. In general, there were medium FertiQol scores for the total sample (M = 69.10, SD = 13.13). It should be noted that this is the calculation of the total of the modules, including those referring to treatment, which were only completed by the women in the Treatment group. Significant differences were also found both in FertiQol Core (where the items assessing tolerability and the treatment environment were excluded) and in the Total FertiQol Module (the sum of Core and Treatment). Table 3 shows the values obtained under both assumptions for each of the groups:

The association between the Mind–Body subscale and the Thinness factor was negative (*r* = −0.216, *p* < 0.01). The Relational subscale showed a positive association with Investment in Appearance (*r* = 0.218, *p* < 0.01), Sexual Fidelity (*r* = 0.212, *p* < 0.05), Relational (*r* = 0.222, *p* < 0.01), and CFNI-Total (*r* = 0.245, *p* < 0.01). Since the participants in the PRE group only had to answer the questions of the FertiQol Core subscales, as they had not yet undergone treatment, the associations between the FertiQol Total score and the CFNI-23 factors were excluded in the interpretation of the results. Finally, significant positive associations were found between the Core value subscale and the Investment in Appearance factor (*r* = 0.179, *p* < 0.05). Finally, Table 4 shows the values obtained between FertiQol subscales and CFNI-23 factors in the Treatment group:

The association between the Emotional subscale and the Relational factor was positive (ρ = 0.194, *p* < 0.05). The Mind–Body subscale showed a significant association with the Relational factor (ρ = 0.157, *p* < 0.05). However, the association between the Mind–body subscale and the Thinness factor was negative (ρ = −0.176, *p* < 0.05). Associations were shown among the Relational subscale and the factors Childcare (ρ = 0.198, *p* = 0.01), Domestic (ρ = 0.161, *p* = 0.05), Sexual Fidelity (ρ = 0.227, *p* = 0.01), and Relational (ρ = 0.18, *p* = 0.05). Nonetheless, the association between the Relational subscale and Thinness factor was negative (ρ = −0.166, *p* = 0.05). The Relational subscale also showed a positive association with CFNI-Total (ρ = 0.205, *p* = 0.01). The association between the Social subscale and the Childcare and Relational factors was positive (ρ = 0.197, *p* = 0.05; ρ = 0.266, *p* = 0.01, respectively). A positive association between the Environment subscale and Childcare was observed (ρ = 0.188, *p* = 0.05). However, the relation between the Environmental subscale and the Thinness factor was negative (ρ = −0.199, *p* = 0.05). The association between the Tolerability subscale and Thinness was negative (ρ = −0.175, *p* = 0.05). The FertiQol Core showed a positive association with the Relational factor (ρ = 0.284, *p* = 0.01); by contrast, its association with Thinness factor was negative (ρ = −0.189, *p* = 0.05). Lastly, the association of FertiQol-Total with the Relational factor was positive (ρ = 0.263, *p* = 0.01), and its association with the Thinness factor was negative (ρ = −0.227, *p* = 0.01).

#### 3.2.1. Clinical Variables and FertiQol Results

This section presents the results of Fertility and Quality of Life (FertiQol) and clinical variables.

#### 3.2.2. Diagnosis

One-way ANOVA for the FertiQol sample and type of diagnoses in PRE showed that inter-group variability in the score was not significantly higher than intra-group variability; *F*(4, 85) = 1.686, *p* = 0.161. For the Treatment group, intra-group heterogeneity was higher than inter-group heterogeneity; *F*(3, 129) = 0.821, *p* = 0.484.

#### 3.2.3. Previous Pregnancies

*T*-tests for independent samples were performed and showed that women with previous pregnancies reported higher quality of life in the PRE group (M = 72.03, SD = 11.49) than in the Treatment group (M = 65.58, SD = 13.69); *t*(54) = 1.849, *p* = 0.069.

#### 3.2.4. Full-Term Pregnancies

Women without a previous full-term pregnancy reported significantly higher quality of life in the PRE group (M = 71.5, SD = 12.49) than in the Treatment group (M = 67.23, SD = 13.79); *t*(197) = 2.205, *p* = 0.029.

#### 3.2.5. Abortions

Women with previous abortions reported significantly higher quality of life in the PRE group (M = 71.76, SD = 11.37) than in the Treatment group (M = 63.15, SD = 13.65); *t*(45) = 2.291, *p* = 0.026. Differences were also found in the Treatment group between women with previous abortions (M = 63.15, SD = 13.65) and women without previous abortions (M = 68.65, 13.52); *t*(127) =1.874, *p* = 0.063. 

#### 3.2.6. Number of Treatment Cycles

The association between FertiQol score and the number of treatment cycles for women in the Treatment group was negative (ρ = −0.27, *p* = 0.002), suggesting that, overall, as the number of cycles increases, quality of life decreases.

### 3.3. Clinical Variables and CFNI-23 Results

This section presents the results of the Female Gender Norms Conformity Inventory (CFNI-23) and clinical variables.

#### 3.3.1. Diagnosis

No differences in means were found in the PRE and Treatment groups according to diagnostic factors: *F*(4, 132) = 0.272, *p* = 0.895; *F*(4, 167) = 0.371, *p* = 0.829, respectively. 

#### 3.3.2. Previous Pregnancies

No significant differences were identified in the scores of previously pregnant and not previously pregnant women in both sample groups.

#### 3.3.3. Full-Term Pregnancies

Women with a previous full-term pregnancy reported significantly lower CFNI-23 mean score in the PRE group (M = 47.56, SD = 4.36) than in the Treatment group (M = 52.22, SD = 4.18); *t*(16) =2.318, *p* = 0.034. Significant differences were also found in the Treatment group between women with previous full-term pregnancy (M = 52.22, SD = 4.18) and women without previous full-term pregnancy (M = 46.28, SD = 6.7); *t*(166) = 2.630, *p* = 0.009.

#### 3.3.4. Abortions

No significant differences were found in the means of CFNI-23 between the PRE and Treatment groups. In addition, no significant differences were detected between the means of the groups of women with and without previous abortions.

#### 3.3.5. Number of Treatment Cycles

No significant association was found between the number of treatment cycles and the CFNI-23 score (ρ = −0.06, *p* = 0.412).

## 4. Discussion

This study contributes to the knowledge of how ART and infertility itself interfere with or modulate quality of life and the impact of gender on health processes. In this regard, it represents progress in the in-depth understanding of the relationships between existing conventional gender norms and the impossibility of traditional parenting that may modulate mental health in expectant mothers in perinatal stages. In line with previous studies, the present study also showed that some clinical factors—such as previous pregnancies—interfered with the means of gender norm conformity in women undergoing treatment [18,28,29].

Previous studies have already demonstrated the impact of assisted reproduction treatments on the quality of life of infertile women during and after treatment [28,30]. In this regard, it is interesting to determine how, in addition to QofL, the degree of gender conformity is influenced during treatment. The results suggest that further research is needed on precise instruments that can measure the impact of adherence to gender norms in assisted reproduction processes. The results showed a strong relationship between self-perceived quality of life and the degree of adherence to gender norms. Although no significant differences were found between the groups in the total scores, significant data were obtained between the subscales and factors that compose the FertiQol and CFNI-23 instruments. The aspects related to romantic relationships and thinness and their relationship with quality of life in the relational subscale stand out, especially in the sample of women in treatment who presented lower levels of quality of life related to these factors. These factors are closely linked to mental illness, such as postpartum depression, anxiety, or other [29,31]. This confirms the need for health and follow-up itineraries that coordinate ART Units with mental health, reproductive, and primary care staff.

The limitations of the study were the absence of previous studies using the CFNI-23 questionnaire on populations receiving ART and the availability of clinical data collected during the COVID-19 pandemic period, which also made it difficult to follow up with the women who attended the assisted reproduction unit, preventing the performance of a long-term study. The present study analysed two socio-demographically and clinically similar groups, but they were independent groups. It was not a longitudinal study. On the other hand, the questionnaires used are self-administered. The use of self-administered questionnaires may decrease the reliability of some measurements.

Missing data have also been an important issue in the study, specifically for the statistical analysis. The heterogeneous presence of missing data on the most relevant variables has discouraged the implementation of multiple regression analyses to fit the effect of different clinical and socio-demographic variables on quality of life and gender conformity scores. Future research should address this question by designing prospective studies aiming to estimate the impact of these variables on women receiving ART.

Future research is also needed to assess the impact of gender norms on other groups such as LQTBIQ+ who receive assisted reproductive technologies, during and after treatment, in populations that have achieved a full-term pregnancy, and in populations without such an outcome. Finally, it would be beneficial to develop longitudinal studies to evaluate the relationship between the variables of quality of life and gender in the ART user population at two different times—pretreatment and post-treatment—especially in those who finally obtained a birth. This is in addition to the analysis of possible differences in QofL and the degree of conformity to gender norms among members of couples with fertility problems using ART. The prevalence or manifestations of mental illnesses such as postpartum depression and others in the same population could also be investigated. 

## 5. Conclusions

Assisted reproductive technologies modulate the QofL of infertile individuals. In addition, there is a relationship between QofL and the degree of conformity to gender norms in infertile individuals. Therefore, the relationship between both variables is of interest to the scientific community. The results obtained indicate that it is recommended to continue research on these aspects and to consider them as possible risks for the mental health of patients from the initiation of reproductive treatment. Knowledge of these aspects may help health professionals to improve and adapt care and assistance during the infertility process. Furthermore, it is recommended to design studies that delve deeper into these variables and their impact during pregnancy and puerperium after ART. Only in this way will it be possible to generate preventive intervention strategies that address all possible risk factors for mothers undergoing ART. Future research should also consider conformity to gender norms and analyse their role and the adaptive responses of individuals to possible contingencies during ART and after pregnancy. Mental health before and after health processes and pregnancy is a relevant public health issue, as it will determine future relationships between family members and offspring. However, there are still knowledge gaps on how gender norms interfere with the mental health of mothers who have undergone ART.

## Figures and Tables

**Table 1:** Descriptive characteristics of the sample.

	Total (*n* = 438)	PRE (*n* = 256)	Treatment (*n* = 182)	Differences
**Age (** **Mean and SD)**	34.85 (3.45)	34.53 (3.34)	35.31 (3.57)	*p* = 0.020 *
**Nationality (*n* and %)**				
Spain	385 (88.3)	224 (87.5)	161 (89.4)	*p* = 0.534
Europe	25 (5.7)	15 (5.9)	10 (5.6)	*p* = 0.893
Others	26 (6.0)	17 (6.6)	9 (5.0)	*p* = 0.476
**Marital Status**				
Single (cohabiting)	144 (33)	104 (40.6)	40 (22.1)	*p* < 0.001 **
Married	199 (45.5)	79 (30.9)	120 (66.3)	*p* < 0.001 **
Civil partner	93 (21.3)	73 (28.5)	20 (11.0)	*p* < 0.001 **
**Educational level**				
Unfinished primary	9 (2.1)	7 (2.7)	2 (1.1)	*p* = 0.25
Primary studies	65 (15.0)	38 (14.8)	27 (15.2)	*p* = 1
Secondary studies	153 (35.3)	84 (32.8)	69 (38.8)	*p* = 0.223
Higher education	206 (47.5)	127 (49.6)	79 (44.4)	*p* = 0.357
**Employment Status**				
Employee	343 (79.2)	194 (75.8)	149 (84.2)	*p* = 0.046 *
Student	4 (0.9)	3 (1.2)	1 (0.6)	*p* = 0.89
Homemaker	12 (2.8)	10 (3.9)	2 (1.1)	*p* = 0.152
Disability	3 (0.7)	3 (1.2)	0 (0.0)	*p* = 0.392
Unemployed	61 (14.1)	40 (15.6)	21 (11.9)	*p* = 0.334
Other	10 (2.3)	6 (2.3)	4 (2.3)	*p* = 1

* *p* < 0.05; ** *p* < 0.01.

**Table 2:** Differences between groups on the FertiQol and CFNI-23 * questionnaires.

Total Scales and Subscales	PRE	Treatment	Difference
	Mean (SD)	Mean (SD)	
*FertiQol*			
Emotional	74.22 (18.4)	70.06 (19.40)	*t*(420) = 2.236, *p* = 0.026
Mind–body	74.7 (18.11)	65.97 (21.64)	*t*(330.517) = 4.391, *p* < 0.001 *
Relational	80.42 (14.34)	79.02 (15.36)	*t*(425) = 0.962, *p* = 0.337
Social	78.04 (16.23)	74.61 (16.48)	*t*(422) = 2.124, *p* = 0.034
Environment	58.53 (18.63)	53.22 (21.15)	*t*(255) = 2.063, *p* = 0.04
Tolerability	68.15 (23.11)	59.43 (24.27)	*t*(262) = 2.911, *p* = 0.004
Core	76.7 (13.24)	72.49 (14.68)	*t*(397) = 3.045, *p* = 0.002
FertiQol-Total	71.44 (12.27)	67.44 (13.51)	*t*(225) = 2.266, *p* = 0.024
*CFNI-23*			
Childcare	10.12 (1.90)	9.53 (2.41)	*t*(321.525) = 2.461, *p* = 0.014 *
Appearance	4.69 (2.41)	5.05 (2.56)	*t*(325) = 1.277, *p* = 0.202
Domestic	7.30 (1.62)	7.43 (1.59)	*t*(326) = 0.714, *p* = 0.476
Sexual fidelity	9.95 (2.12)	9.68 (2.24)	*t*(325) = 1.091, *p* = 0.276
Relational	6.65 (2.05)	6.64 (2.05)	*t*(322) = 0.02, *p* = 0.984
Thinness	3.73 (2.90)	4.11 (2.70)	*t*(325) = 1.208, *p* = 0.228
Modesty	4.08 (2.15)	4.15 (2.03)	*t*(324) = 0.298, *p* = 0.766
CFNI-23-Total	46.53 (6.56)	46.57 (6.79)	*t*(314) = 0.05, *p* = 0.96

* Welch’s *t*-test.

**Table 3:** Pearson’s correlations between the FertiQol * subscales and CFNI-23 factors in the PRE group.

Subscales	F1	F2	F3	F4	F5	F6	F7	CFNI-Total
Emotional	−0.063	0.082	0.023	−0.106	−0.01	−0.151	−0.003	−0.095
Mind–body	−0.002	0.083	0.036	−0.139	0.094	−0.216 **	0.025	−0.076
Relational	0.149	0.218 **	0.039	0.212 *	0.222 **	0.009	−0.066	0.245 **
Social	0.054	0.143	0.078	0.000	0.096	0.009	0.058	0.122
Environment	0.183	0.154	−0.055	0.065	0.005	0.015	−0.035	0.158
Tolerability	−0.135	0.141	−0.124	−0.157	0.092	−0.269 *	−0.033	−0.158
Core	0.035	0.179 *	0.066	−0.005	0.122	−0.118	0.021	0.066
FertiQol-Total	−0.008	0.198	−0.052	−0.067	0.168	−0.259 *	0.065	0.006

Note. F1 = Care for children, F2 = Investment in appearance, F3 = Domestic, F4 = Sexual fidelity, F5 = Relational, F6 = Thinness, F7 = Modesty; * *p* < 0.05; ** *p* < 0.01.

**Table 4:** Spearman’s correlations between FertiQol subscales and CFNI-23 factors in the Treatment group.

Subscales	F1	F2	F3	F4	F5	F6	F7	CFNI-Total
Emotional	0.031	−0.003	0.125	−0.034	0.194 *	−0.139	0.021	0.049
Mind–body	0.017	−0.111	0.038	−0.021	0.157 *	−0.176 *	−0.09	−0.056
Relational	0.198 **	0.059	0.161 *	0.227 **	0.18 *	−0.166 *	−0.048	0.205 **
Social	0.197 **	0.041	0.043	0.038	0.266 **	−0.125	−0.079	0.127
Environment	0.188 *	0.126	0.045	−0.032	0.044	−0.199 *	−0.005	0.07
Tolerability	0.109	−0.036	0.032	0.019	0.098	−0.175 *	−0.083	−0.014
Core	0.137	−0.007	0.126	0.039	0.284 **	−0.189 *	−0.07	0.099
FertiQol-Total	0.165	0.027	0.089	0.011	0.263 **	−0.227 **	−0.045	0.084

Note. F1 = Care for children, F2 = Investment in appearance, F3 = Domestic, F4 = Sexual fidelity, F5 = Relational, F6 = Thinness, F7 = Modesty. * *p* < 0.05; ** *p* < 0.01.

## Data Availability

Bueno-Sánchez, Lidia (2022), “Dataset_FertiQol_CFNI”, Mendeley Data, V1, doi:10.17632/shyszxprwm.1.

## References

[1] Calhaz-Jorge C., De Geyter C.H., Kupka M.S., Wyns C., Mocanu E., Motrenko T., Scaravelli G., Smeenk J., Vidakovic S., Goossens V. (2020). Survey on ART and IUI: Legislation, regulation, funding and registries in European countries: The European IVF-monitoring Consortium (EIM) for the European Society of Human Reproduction and Embryology (ESHRE). Hum. Reprod. Open.

[2] Salama M., Isachenko V., Isachenko E., Rahimi G., Mallmann P., Westphal L.M., Inhorn M.C., Patrizio P. (2018). Cross border reproductive care (CBRC): A growing global phenomenon with multidimensional implications (a systematic and critical review). J. Assist. Reprod. Genet..

[3] Ying L., Wu L.H., Loke A.Y. (2016). The effects of psychosocial interventions on the mental health, pregnancy rates, and marital function of infertile couples undergoing in vitro fertilization: A systematic review. J. Assist. Reprod. Gene.

[4] Ockhuijsen H., Van den Hoogen A., Eijkemans M., Macklon N., Boivin J. (2014). The impact of a self-administered coping intervention on emotional well-being in women awaiting the outcome of IVF treatment: A randomized controlled trial. Hum. Reprod..

[5] Bolvin J., Lancastle D. (2010). Medical Waiting Periods: Imminence, Emotions and Coping. Women’s Health.

[6] Cserepes R.E., Bugán A., Korösi T., Toth B., Rösner S., Strowitzki T., Wischmann T. (2014). Infertility specific quality of life and gender role attitudes in German and Hungarian involuntary childless couples. Geburtshilfe Frauenheilkd.

[7] Luk H.-K.B., Loke A. (2015). The impact of infertility on the psychological well-being, marital relationships, sexual relationships, and quality of life of couples: A systematic review. J. Sex Marital Ther..

[8] Verhaak C., Smeenk J., Kremer J., Kraaimaat F. (2005). A longitudinal, prospective study on emotional adjustment before, during and after consecutive fertility treatment cycles. Hum. Reprod..

[9] Verhaak C., Smeenk J., Evers A.W.M., Kremer J.A.M., Kraaimaat F.W., Braat D.D.M. (2007). Women’s emotional adjustment to IVF: A systematic review of 25 years of research. Hum. Reprod..

[10] World Health Organization. https://www.who.int/health-topics/gender#tab=tab_1.

[11] Hunt C.J., Christopher J., Gonsalkorale K., Carnaghi A. (2015). Feminine role norms among Australian and Italian women: A cross-cultural comparison. Sex Roles.

[12] Sánchez-López M.P., Limiñana R.M. (2017). Health from a gender perspective: The state of the art. The Psychology of Gender and Health.

[13] Esteban-Gonzalo L., Manso-Martínez M., Botín-González P., Manchado-Simal B., Rodrigo-De-Frutos R.M., González-Pascual J.L. (2019). The relationship between conformity to male and female gender norms and depression during pregnancy. Arch. Women Ment. Health.

[14] Aparicio-García M., Fernández-Castilla B., Giménez-Páez M.A., Piris-Cava E., Fernández-Quijano I. (2018). Influence of feminine gender norms in symptoms of anxiety in the Spanish context. Ansiedad Estrés.

[15] Zentner M., Von Aufsses C. (2020). Is being gender nonconforming distressing? It depends where you live: Gender equality across 15 nations predicts how much gender nonconformity is related to self-esteem. Psychol. Med..

[16] Grunberg P., Miner S., Zelkowitz P. (2022). Infertility and perceived stress: The role of identity concerns in treatment-seeking men and women. Hum. Fertil..

[17] Morray E.B. (2010). The Shared Burden of Infertility: Gender Role Conformity as a Predictor of Infertility-Related Distress and Relational Health in Couples Undergoing Treatment for Infertility. Ph.D. Thesis.

[18] Abbey A., Andrews F.M., Halman L.J. (1994). Psychosocial predictors of life quality: How are they affected by infertility, gender, and parenthood?. J. Fam. Issues.

[19] García-Quintáns L. (2017). Análisis de Estilos de Personalidad Género y Salud en Parejas que Presentan Problemas de Fertilidad. Unpublished Thesis.

[20] Daniluk J.C., Tench E. (2007). Long-term adjustment of infertile couples following unsuccessful medical intervention. J. Couns. Dev..

[21] Abbey A., Andrew F.M., Halman L.J. (1991). Gender’s role in responses to infertility. Psychol. Women Q..

[22] European Regulation (EU) 2016/679 Consolidated Text of the European Parliament and of the Council of 27 April 2016 on the Protection of Natural Persons with Regard to the Processing of Personal Data and on the Free Movement of such Data, and Repealing Directive 95/46/EC (General Data Protection Regulation) (Text with EEA Relevance). https://eur-lex.europa.eu/legal-content/EN/TXT/?uri=CELEX%3A02016R0679-20160504.

[23] Boivin J., Takefman J., Braverman A. (2011). The Fertility Quality of Life (FertiQoL) tool: Development and general psychometric properties. Fertil. Steril..

[24] Fertility Quality of Life tool. Download FertiQol. http://sites.cardiff.ac.uk/fertiqol/download/.

[25] Aarts J., Van Empel W.M., Boivin J., Nelen W.L., Kremer J.A.M., Verhaak C.M. (2011). Relationship between quality of life and distress in infertility: A validation study of the Dutch FertiQoL. Hum. Reprod..

[26] Mahalik J.R., Morray E.B., Coonerty-Femiano A., Ludlow L.H., Slattery S.M., Smiler A. (2005). Development of the Conformity to Feminine Norms Inventory. Sex Roles.

[27] Garcia-Cano A., Martinez C. (2017). Spanish adaptation of the Conformity to Feminine Norms Inventory-45. UARICHA Rev. Psicol..

[28] Huppelschoten A.G., Dongen A.J.C.M., Werhaak C.M., Smeenk J., Kremer J.A.M., Nelen W.L.D.M. (2013). Differences in quality of life and emotional status between infertile women and their partners. Hum. Reprod..

[29] Przybyła-Basista H., Kwiecińska E., Ilska M. (2020). Body Acceptance by Pregnant Women and Their Attitudes toward Pregnancy and Maternity as Predictors of Prenatal Depression. Int. J. Environ. Res. Public Health.

[30] Verhaak C.M., Lintsen A.M.E., Evers A.W.M., Braat D.D.M. (2010). Who is at risk of emotional problems and how do you know? Screening of women going for IVF treatment. Hum. Reprod..

[31] Toribio-Caballero S.V., Cardenal-Hernáez A., Ávila A., Ovejero M. (2022). Roles De género Y Salud Mental En Las Mujeres: Su Influencia En La Demanda De atención psicológica. Annals Psycholog..

